# *Lycium barbarum* Extracts Extend Lifespan and Alleviate Proteotoxicity in *Caenorhabditis elegans*

**DOI:** 10.3389/fnut.2021.815947

**Published:** 2022-01-12

**Authors:** Haitao Zhou, Shanshan Ding, Chuanxin Sun, Jiahui Fu, Dong Yang, Xi'e Wang, Chih-chen Wang, Lei Wang

**Affiliations:** ^1^National Laboratory of Biomacromolecules, CAS Center for Excellence in Biomacromolecules, Institute of Biophysics, Chinese Academy of Sciences (CAS), Beijing, China; ^2^Central Laboratory, Luoyang Central Hospital Affiliated to Zhengzhou University, Luoyang, China; ^3^Beijing Key Laboratory of Functional Food From Plant Resources, College of Food Science and Nutritional Engineering, China Agricultural University, Beijing, China; ^4^College of Life Sciences, University of Chinese Academy of Sciences, Beijing, China

**Keywords:** *Lycium barbarum*, aging, lifespan, neurodegenerative diseases, *hsf-1*, *sir-2.1*

## Abstract

*Lycium barbarum* berry (Ningxia Gouqi, *Fructus lycii*, goji berry, or wolfberry), as a traditional Chinese herb, was recorded beneficial for longevity in traditional Chinese medical scriptures and currently is a natural dietary supplement worldwide. However, under modern experimental conditions, the longevity effect of *L. barbarum* berry and the underlying mechanisms have been less studied. Here, we reported that total water extracts of *L. barbarum* berry (LBE), which contains 22% polysaccharides and other components, such as anthocyanins, extended the lifespan of *Caenorhabditis elegans* without side effects on worm fertility and pharyngeal pumping. Interestingly, we found that the lifespan extension effect was more prominent in worms with shorter mean lifespan as compared to those with longer mean lifespan. Furthermore, we showed that the lifespan extension effect of LBE depended on deacetylase *sir-2.1*. Remarkably, LBE rescued heat shock transcription factor-1 (*hsf-1*) deficiency in wild-type worms with different mean lifespans, and this effect also depended on *sir-2.1*. In addition, we found that LBE extended lifespan and alleviated toxic protein aggregation in neurodegenerative worms with *hsf-1* deficiency. Our study suggested that LBE may be a potential antiaging natural dietary supplement especially to individuals with malnutrition or chronic diseases and a potential therapeutic agent for neurodegenerative diseases characterized by *hsf-1* deficiency.

## Introduction

*Lycium barbarum* berry (Ningxia Gouqi, Goji, goji berry, or wolfberry) is a traditional Chinese herb and functional food that has been used for thousands of years worldwide (1). Current pharmacological research shows that it is composed of *L. barbarum* polysaccharides (LBP), flavonoids, carotenoids, betaine, zeaxanthin, physalien, trace minerals, and other substances (2). As for the effect of *L. barbarum* berry, traditional Chinese medical scriptures such as “Shen Nong Ben Cao Jing” and “Ben Cao Gang Mu” (Compendium of Materia Medica) recorded that it possesses the function of nourishing liver and kidney, strengthening muscles and bones, improving eyesight, and extending lifespan. However, the lifespan extension effect of *L. barbarum* berry has been rarely studied under experimental conditions and lacks scientific evidences. Additionally, it is not clear whether *L. barbarum* berry has different effects on individuals living under different conditions. Thus, it is necessary to use animal models cultured under different experimental conditions to study the antiaging effect of *L. barbarum* berry.

The function of heat shock transcription factor-1 (*hsf*-1), the ortholog of *Caenorhabditis elegans hsf*-1, is to regulate the expression of heat shock proteins to maintain proteostasis (3) and cytoskeletal integrity (4), which is beneficial for stress resistance and longevity. Activation of *hsf*-1 or overexpressing chaperones can increase longevity and stress resistance (5, 6), whereas deficiency or deregulation of *hsf*-1 activity is associated with accelerated aging phenotypes and neurodegenerative diseases, such as Alzheimer's disease, Huntington's disease, amyotrophic lateral sclerosis, and Parkinson's disease (7, 8). Pharmacological approaches for maintaining or enhancing proteostasis and cytoskeletal integrity by rescuing *hsf*-1 deficiency are promising and potential strategies for extending lifespan and neurodegenerative disease interventions (9, 10).

Heat shock transcription factor-1 activity is regulated by acetylation at critical residues, which attenuates its DNA-binding activity and reduces the expression of molecular chaperons including Hsp70 and Hsp90 (11, 12). Conversely, deacetylation of *hsf*-1 by the deacetylase SIRT1, the ortholog of *C. elegans* sir-2.1, promotes the binding of *hsf*-1 to heat shock elements, which activates heat shock responses to maintain proteostasis and longevity (11).

In this study, we found that total water extracts of *L. barbarum* berry (LBE) mediated longevity negatively correlated with mean lifespan in *C. elegans*. In worms with shorter mean lifespan, LBE showed a better antiaging effect, depending on *sir-2.1*. Additionally, LBE rescued *hsf-1* deficiency in wild-type worms, also in a *sir-2.1-*dependent manner. Moreover, LBE extended lifespan and alleviated toxic protein aggregation in neurodegenerative worms with *hsf-1* deficiency.

## Materials and Methods

### LBP Preparation

The LBP from *L. barbarum berries* was prepared as described (13). The dried fruits of *L. barbarum* were crushed by a grinder. Then, the powder was extracted with 55°C water and assisted with enzymes (6% cellulase, 0.1% papain, and 2% amylase) under stirring for 1 h, followed by enzyme inactivation by heating the solution at 100°C. After filtered, the extracted solution was concentrated and dialyzed against running water by dialysis membrane (molecular weight cutoff = 3500 Da) for 3 days. The retentate was added five volumes of 95% ethanol for overnight. The precipitate was washed with acetone and absolute ethanol alternately for three times and then freeze-dried in vacuum to yield the crude polysaccharide LBP.

### LBE Preparation and Determination of Principal Components

#### LBE Preparation

The LBE was prepared as described (14). *L. barbarum* berries were obtained from Zhongning County of Ningxia Hui Autonomous Region in China and identified by Dr. Changcai Bai (Ningxia Medical University). The dried berries (100 g) were soaked in water (1 l) at room temperature after being washed five times. Then, they were decocted with neutral water (2 l) at boiling temperature two times for 2.0 and 1.5 h, respectively. The combined concentrated decoctions were filtered through a hollow fiber membrane and evaporated under vacuum (1 kPa) at 45°C to remove the water and obtain the concentrates. The main component of the *L. barbarum* extract was water-soluble polysaccharides, also with flavonoids, carotenoids, and anthocyanins, according to the published papers which used the similar protocol to extract *L. barbarum* (15, 16). The constant volume method was used to obtain 100 ml of concentrate (concentration defined as 1 g/ml), which was diluted to 200 mg/ml for experiments or stored at −20°C.

#### Determination of Polysaccharide Content

The method is principally the same as previously described (17). 5 μl of LBE (100 mg/ml) was added to 1,995 μl of distilled water as experimental group. Next, 1 ml of 6% phenol solution and 5 ml of concentrated sulfuric acid was added to the abovementioned tubes and then incubated at room temperature for 10 min. After mixing, the mixture was further incubated for 20 min. The absorbance at 490 nm (A490) of these solutions was measured with a spectrophotometer (DS-11 FX+, DeNovix, Wilmington, Germany). The standard curve was drawn with glucose standard solution. The polysaccharide concentration in the solution was derived with the standard curve.

#### Determination of Anthocyanin Content

About 20 μl of LBE (100 mg/ml) was added to 980 μl of hydrochloric acid solution (pH 1.0) and sodium acetic buffer (pH 4.5), respectively. Then, the abovementioned two mixed solutions were incubated in dark at room temperature for 30 min. Hydrochloric acid solution (pH 1.0) and sodium acetic buffer (pH 4.5) were used as a control, respectively. The absorbance at 526 nm (A_526_) and 700 nm (A_700_) of these two solutions was measured with a spectrophotometer (DS-11 FX+, DeNovix, Wilmington, Germany). The anthocyanin content was calculated according to previous studies (18). The calculation formula was as follows:

The anthocyanin content (mgC3G/ml) = (ΔA^*^Mw^*^DF)/(ε^*^l) Here:

ΔA = (A520–A700) pH1.0 – (A520–A700) pH4.5. Mw represents the molecular weight of cyanidin-3-O-glucoside, which equals 449.2. DF represents the dilution factor. ε represents the molar absorptivity equaling 26900, and l represents the path length, which was 1 cm in this experiment.

### *C. elegans* Strains and Bacterial Clones

The nematode strains wild type (N2), SJ17 *xbp-1(zc12)*, and VC199 *sir-2.1(ok434)*, and also bacterial clones HT115(DE3) RNase III-deficient *Escherichia coli* transformed by empty vector L4440 or vector carrying vehicle, *hsf-1, daf-16*, or *sir-2.1* RNAi fragment were the gifts from Hong Zhang (Institute of Biophysics, Chinese Academy of Sciences). Strains VC199 *sir-2.1(ok434)* and CF1038 *daf-16(mu86)* were the gifts from Xiaochen Wang (Institute of Biophysics, Chinese Academy of Sciences). Strain PS3551 *hsf-1(sy441)*, GMC101 (*unc-54p::A*β*1-42::unc-54 3'-UTR*), and NL5901 (*unc-54p::*α*-synuclein::YFP)* were the gifts from Ye Tian (Institute of Genetics and Developmental Biology, Chinese Academy of Sciences).

### Worm Culture Conditions and Treatment

Nematode growth media (NGM) agar plates were prepared as described (19, 20). Worms were cultured at 20°C on NGM seeded with *E. coli* OP50 from an overnight culture. When needed, LBP or LBE were added into molten agar (19, 21) to the final indicated concentration from 60 mg/ml or 200 mg/ml stock solution in water, respectively. Control groups were added with an equivalent amount of water.

### RNAi Assays

RNAi assays were performed as described (22). Briefly, a single clone of HT115 bacteria containing vehicle or specific RNAi fragment plasmids was picked from LB plates with 50 μg/ml of ampicillin and 5 μg/ml of tetracycline and cultured in LB liquid medium with 50 μg/ml of ampicillin for 6–8 h. Then, 50 μl of culture onto 35-mm NGM plates with 50 μg/ml of ampicillin and 1 mM of isopropyl β-D-thiogalactopyranoside was spotted. Seeded plates were placed at room temperature to dry and continued overnight to induce the expression of dsRNA.

### Lifespan Assays

Lifespan assays were performed at 20°C as described (20), unless otherwise indicated. To make worms with different mean lifespans, we divided the worms into two conditions. The worms that had been continuously cultured at optimal growth conditions for at least two generations before synchronization by hatching in M9 for the lifespan assay were defined as a favorable condition group (23). Otherwise, they were defined as an unfavorable condition group. When the plates had a large number of gravid adult hermaphrodites, eggs were collected using alkaline hypochlorite and incubated for 24 h in M9 buffer or on plate without OP50. Then, L1 larvae were placed onto regular or RNAi plates until for L4-stage (day 0).

Lifespan assays were started by transfer of L4 worms at indicated number to plates containing OP50 or RNAi bacteria in the absence or presence of LBE. To prevent progeny production, the surface of 35-mm NGM plates was added with 0.5 ml M9 with 5-fluoro-2′-deoxyuridine (FUdR, 30 μM) as indicated when it was applied. The plates in a biosafety cabinet were blow-dried and utilized immediately or stored at 4°C. If FUdR was not supplemented, worms were transferred to fresh plates daily until day 6 and then transferred every 2–5 days until the end of the experiment. If FUdR was supplemented, worms were transferred to fresh plates every 2–3 days until day 6 and then transferred every 3–5 days until the end of the experiment. FUdR was removed after day 8. Worms were monitored at regular time points every day or every 2nd day. When worms that did not show any movement in touch by platinum wire were scored as dead, worms that cannot be found and showed severe vulva bursting or bagging were censored. All lifespan experiments were repeated at least two times.

### Worm Pharyngeal Pumping Assays

Synchronized adult worms were transferred at L4 to NGM plates in the absence or presence of 5 mg/ml of LBE. Worm pharyngeal pumping assays were started on day 1 of adulthood. For each condition, 5–10 worms were randomly selected from the plates. Pharyngeal pumping rate was tested by counting pumps of the terminal pharyngeal bulb using Leica M205FA dissection scope during a period of 30 s.

### Worm Fertility Assays

About 3–5 synchronized worms were transferred at L4 to NGM plates (1 L4 per plate) in the absence or presence of 5 mg/ml of LBE and allowed to lay eggs. Because of the presence of LBE, the number of eggs is difficult to be observed. Eggs were allowed to hatch into the L1-4 larval stage, and the larvae were counted and transferred every 12 h until day 6. The total number of larvae of each worm is the fertility of the worm.

### Fluorescence Microscopy

About 30 synchronized NL5901 worms at L4 were transferred to plates containing vehicle or specific RNAi bacteria in the absence or presence of 5 mg/ml of LBE. Fluorescence microscopy was performed on day 3 of adulthood using a Leica DMI 4000B microscope and LAS X software. The α-synuclein aggregation in anterior region of worms was classified into three levels according to the degree of aggregation: no puncta, focal puncta, or diffuse puncta.

### Statistical Analysis

The statistical significance in different groups was assessed using Kaplan–Meier log-rank test (lifespan assays), Mann–Whitney *U*-test (fluorescence microscopy of α-synuclein aggregation), Pearson's correlation test (the relationship between longevity effects of LBE and mean lifespan), or unpaired *t*-test (worm pharyngeal pumping and fertility assay). Data were analyzed using the Statistical Package for the Social Sciences (SPSS) 23.0 on Windows 10.0. *p* < 0.05 was considered statistically significant.

## Results

### LBP at 300μg/ml Did Not Prolong Lifespan of Worms Under Favorable or Unfavorable Conditions

**Figure 1:**
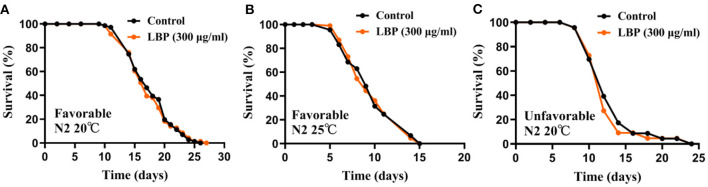
LBP did not prolong the lifespan of worms at either 20 or 25°C. **(A)** Survival curves of N2 worms cultured with vehicle or 300 μg/ml LBP at 20°C under favorable conditions (control: *n* = 71, mean lifespan 17.8 days; 300 μg/ml LBP: *n* = 71, mean lifespan 17.3 days; *p* = 0.864). **(B)** Survival curves of N2 worms cultured with vehicle or 300 μg/ml LBP at 25°C under favorable conditions (control: *n* = 89, mean lifespan 9.7 days; 300 μg/ml LBP: *n* = 108, mean lifespan 9.7 days; *p* = 0.898). **(C)** Survival curves of N2 worms cultured with vehicle or 300 μg/ml LBP at 20°C under unfavorable conditions (control: *n* = 23, mean lifespan 13.0 days; 300 μg/ml LBP: *n* = 22, mean lifespan 12.5 days; *p* = 0.864). The results are representative of at least two independent lifespan experiments. Log-rank test was used to assess significance (see Supplementary Table 1). LBP, *L. barbarum* polysaccharides.

*L. barbarum* polysaccharide is the main active ingredients of *L. barbarum* or LBE. Previous studies showed that 300 μg/ml of LBP was the best concentration for extending the lifespan of N2 worms at 20 or 25°C (+17 and 21%, respectively) (24). Therefore, we first verified the antiaging effect of LBP at 300 μg/ml for worms under favorable conditions (continuously cultured at optimal growth conditions at 20°C for at <2 generations and synchronized by hatching in M9). In our hands, we found that LBP did not prolong the lifespan of worms at 20°C (Figure 1A). In addition, LBP at 300 μg/ml did not prolong the lifespan of worms at 25°C (Figure 1B).

We also tested the antiaging effect of LBP at 300 μg/ml under unfavorable conditions (continuously cultured at optimal growth conditions at 20°C for <2 generations and synchronized by hatching on plate without OP50), in which worms had a shorter mean lifespan. We also found that LBP did not prolong the lifespan of worms at 20°C under unfavorable conditions (Figure 1C). Although we cannot exclude the possibility that different preparation methods of LBP may be one of the reasons for the inconsistency between our results and the previous, our results suggest that LBP alone may be not an effective antiaging nutritional supplements for worms.

### LBE Extends Lifespan of Worms Under Unfavorable Conditions Without Side Effects on Fertility and Pharyngeal Pumping

Since LBP alone has no antiaging effect, we then tested the antiaging effect of LBE. We first measured the concentrations of polysaccharides and anthocyanins in LBE, which are the principal and distinguishing components of *L. barbarum* (15, 16). The results showed that the polysaccharide concentration was 22.03 mg/ml and the anthocyanins concentration was 0.47 μg/ml in 100 mg/ml of LBE, consistent with the polysaccharide and anthocyanin yields in previous reports (25, 26).

**Figure 2:**
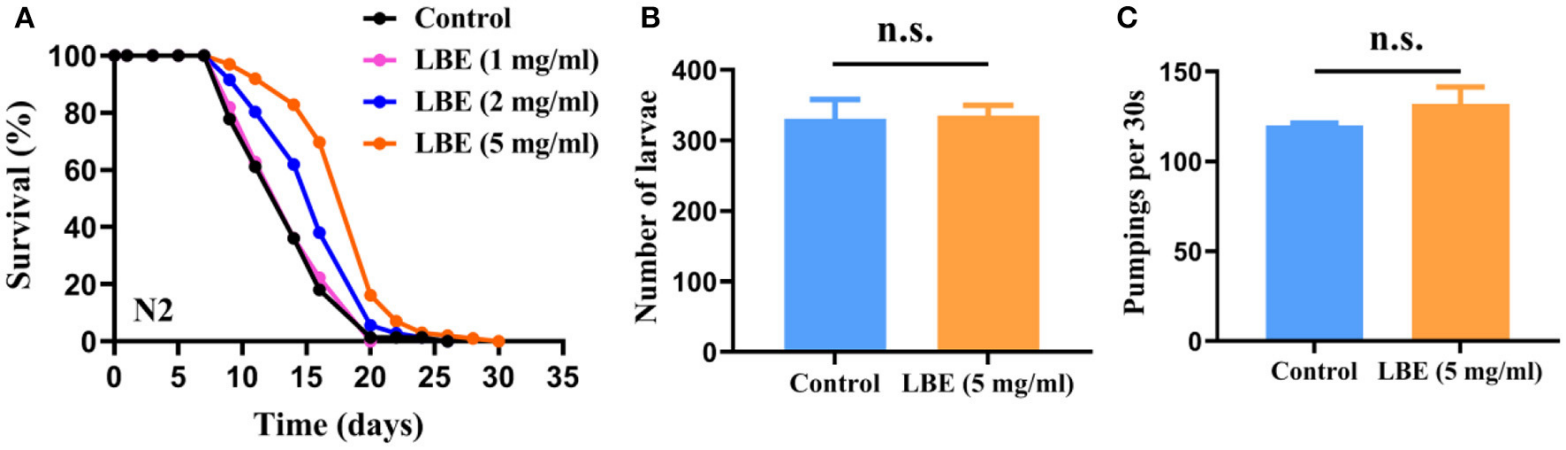
LBE extends the lifespan of *C. elegans* without side effects on fertility and pharyngeal pumping. **(A)** Survival curves of N2 worms cultured with vehicle or 1, 2, and 5 mg/ml LBE (control: *n* = 49, mean lifespan 13.9 days; 1 mg/ml LBE: *n* = 51, mean lifespan 14.1 days, *p* = 0.703 vs. control; 2 mg/ml LBE: *n* = 91, mean lifespan 16.2 days, *p* = 0.002 vs. control; 5 mg/ml LBE: *n* = 68, mean lifespan 18.7 days, *p* < 0.0001 vs. control). **(B)** The number of larvae fertility of worms treated with vehicle (*n* = 4) or 5 mg/ml LBE (*n* = 4). **(C)** The frequency of pharyngeal pumping of worms treated with vehicle (*n* = 5) or 5 mg/ml LBE (*n* = 5). Error bars represent SD. The results are representative of at least two independent experiments. Log-rank test **(A)** or unpaired *t*-test **(B,C)** was used to assess significance. n.s., not significant. LBE, total water extracts of *L. barbarum* berry.

In contrast to the results of LBP under unfavorable conditions, LBE at concentrations of 1, 2, and 5 mg/ml increased the mean lifespan of worms by 4, 16, and 35%, respectively, as compared to the vehicle-treated worms (Figure 2A). The results showed a clear dose-dependent effect on lifespan by LBE. However, LBE at 10 mg/ml had no better antiaging effect than 5 mg/ml (Supplementary Figure 1). Thus, the optimum concentration of LBE for lifespan extension in worms was 5 mg/ml.

Studies have shown that reduction of fertility is often associated with lifespan extension through reallocation of resources to somatic cells. We found that LBE treatment extended the lifespan without affecting the fertility of worms, which even produced slightly more offspring than the vehicle-treated groups (Figure 2B). In addition, dietary restriction by fasting can robustly extend the lifespan of worms. To exclude this possibility, we measured the effect of LBE on pharyngeal pumping of worms. We observed that LBE did not slow down the frequency of pharyngeal pumping (Figure 2C). These findings indicated that LBE did not extend lifespan by reducing fertility or dietary restriction.

### The Longevity Effect of LBE Is Negatively Correlated With the Mean Lifespan Length in Worms

**Figure 3:**
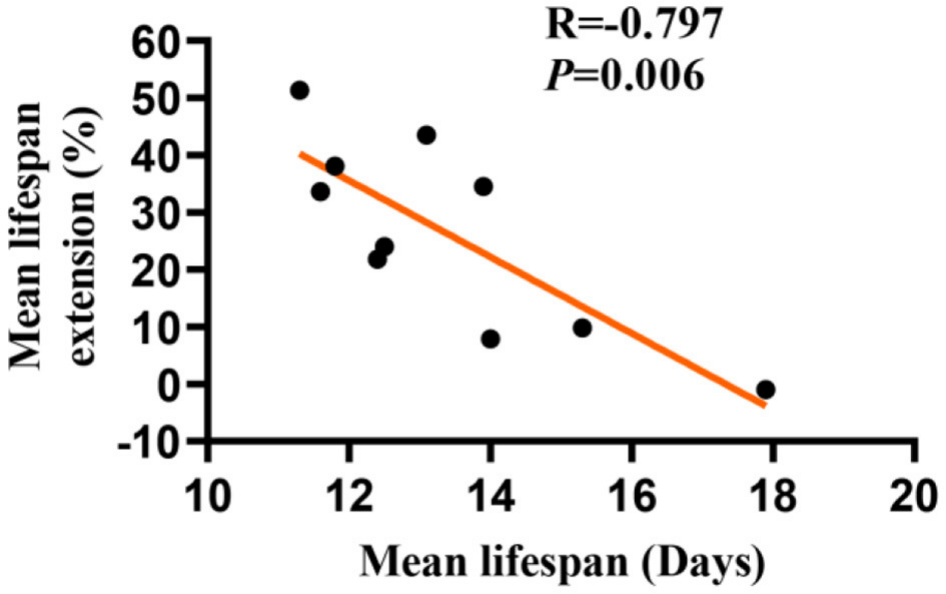
The longevity effects of LBE are negatively correlated with the mean lifespan in *C. elegans*. Lifespan extension effect of LBE at 5 mg/ml was plotted against the mean lifespan of worms cultured under different conditions. Pearson's correlation test was used to assess significance (see also Supplementary Table 3). LBE, total water extracts of *L. barbarum* berry.

Whether the longevity effect of LBE depends on the mean lifespan remains unclear. To address this question, we studied the effect of LBE at 5 mg/ml on the lifespan of *C. elegans*, which were cultured under favorable or unfavorable conditions to induce different mean lifespan. We found that the lifespan extension effect of LBE decreased with the increase of mean lifespan (Figure 3). At the extreme, when the mean lifespan of worms reached 17 days or even longer, LBE had no effect on lifespan extension (Supplementary Figure 2A). Moreover, LBE at the concentration of 1 mg/ml or 2 mg/ml was not effective (Supplementary Figures 2B,C). Taken together, these findings suggested that the longevity effect of LBE is negatively correlated with mean lifespan in *C. elegans*.

### LBE Extends Lifespan Depending on *sir-2.1* Under Unfavorable Conditions

It has been reported that the lifespan of worms can be regulated by several pathways, including heat shock response, energy metabolic homeostasis, insulin/IGF-1-like signaling (IIS), unfolded protein response, and sirtuin signaling. To understand the mechanism of antiaging effect of LBE, nematodes with key protein mutations in these pathways were treated with LBE at 5 mg/ml under unfavorable conditions.

**Figure 4:**
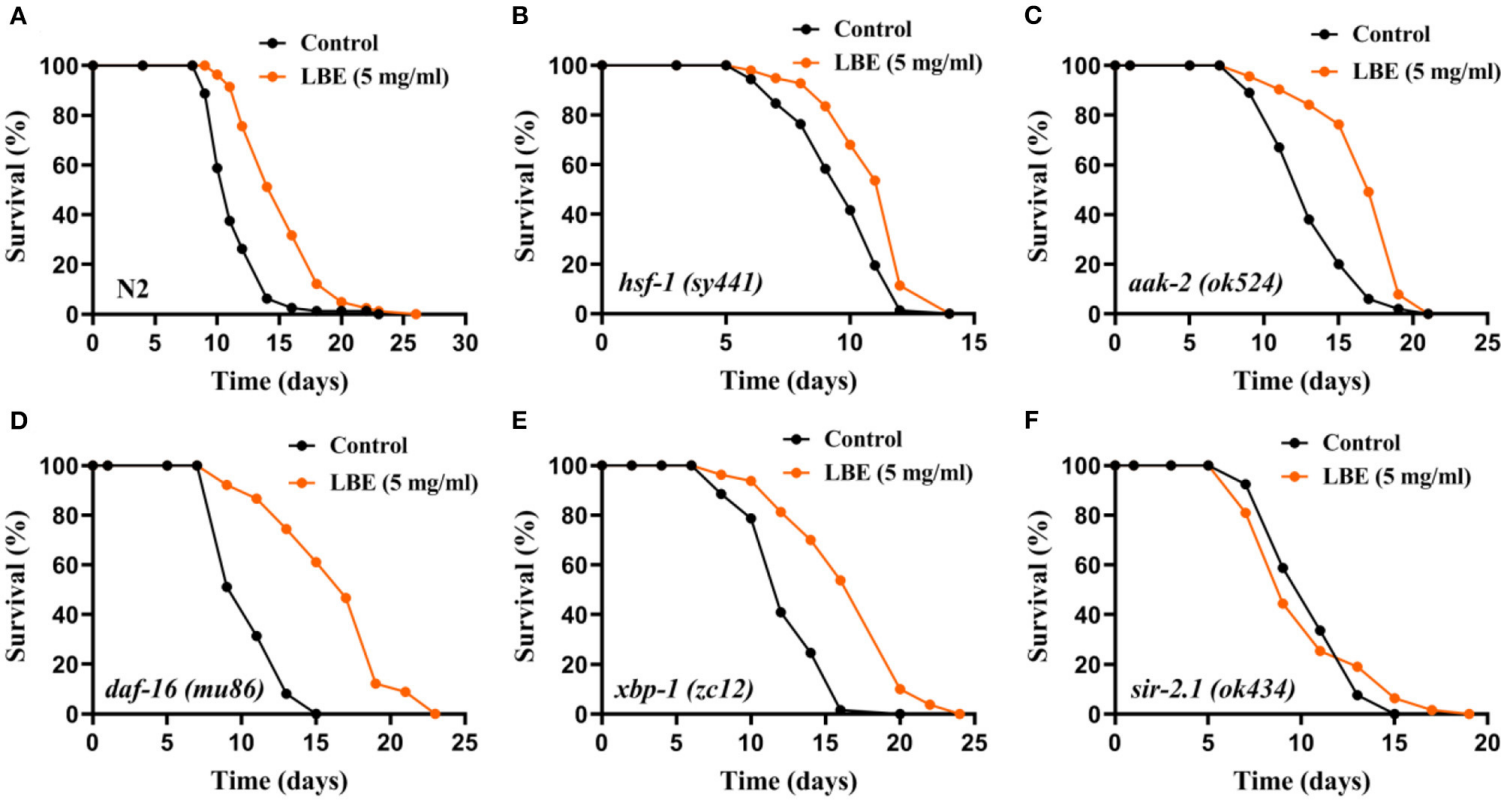
LBE extends lifespan depending on *sir-2.1* under unfavorable conditions. **(A)** Survival curves of N2 worms cultured with vehicle or 5 mg/ml LBE at 20°C (control: *n* = 80, mean lifespan 11.6 days; 5 mg/ml LBE: *n* = 82, mean lifespan 15.5 days; *p* < 0.0001). **(B)** Survival curves of *hsf-1 (sy4421)* worms cultured with vehicle or 5 mg/ml LBE at 20°C (control: *n* = 72, mean lifespan 9.7 days; 5 mg/ml LBE: *n* = 97, mean lifespan 11.1 days; *p* < 0.0001). **(C)** Survival curves of *aak-2 (ok524)* worms cultured with vehicle or 5 mg/ml LBE at 20°C (control: *n* = 100, mean lifespan 13.4 days; 5 mg/ml LBE: *n* = 114, mean lifespan 17.1 days; *p* < 0.0001). **(D)** Survival curves of *daf-16 (mu86)* worms cultured with vehicle or 5 mg/ml LBE at 20°C (control: *n* = 86, mean lifespan 10.8 days; 5 mg/ml LBE: *n* = 90, mean lifespan 16.6 days; *p* < 0.0001). **(E)** Survival curves of *xbp-1 (zc12)* worms cultured with vehicle or 5 mg/ml LBE at 20°C (control: *n* = 61, mean lifespan 12.7 days; 5 mg/ml LBE: *n* = 80, mean lifespan 17.3 days; *p* < 0.0001). **(F)** Survival curves of *sir-2.1 (ok434)* worms cultured with vehicle or 5 mg/ml LBE at 20°C (control: *n* = 119, mean lifespan 10.8 days; 5 mg/ml LBE: *n* = 63, mean lifespan 10.6 days; *p* = 0.981). All the results are representative of at least two independent lifespan experiments. Log-rank test was used to assess significance (see also Supplementary Table 4). LBE, total water extracts of *L. barbarum* berry.

The results showed that the mean lifespan of N2 and mutant nematodes including *hsf-1* (heat shock response), *aak-2* (energy metabolism), *daf-16* (IIS), and *xbp-1* (unfolded protein response) was extended by LBE, except for *sir-2.1* (sirtuin signaling) (Figure 4). As compared to N2 worms (+34%, Figure 4A), LBE had less lifespan extension effect on mutant strains *hsf-1 (sy441)* and *aak-2 (ok524)* (+14 and 27%, respectively; Figures 4B,C). The results indicated that the effect of LBE on lifespan is partially dependent on *aak-2* and *hsf-1*. LBE had a similar lifespan extension effect on mutant strains *daf-16 (mu86)* and *xbp-1 (zc12)*, as compared to N2 worms (+54 and 36%, respectively; Figures 4D,E). However, when the mutant strain *sir-2.1 (ok434)* was treated with LBE, no lifespan extension was observed as compared to vehicle-treated groups (Figure 4F). Altogether, our results suggested that the effect of LBE on lifespan under unfavorable conditions depends on *sir-2.1*.

### LBE Rescues *hsf-1* Deficiency in Worms Under Favorable Conditions

**Figure 5:**
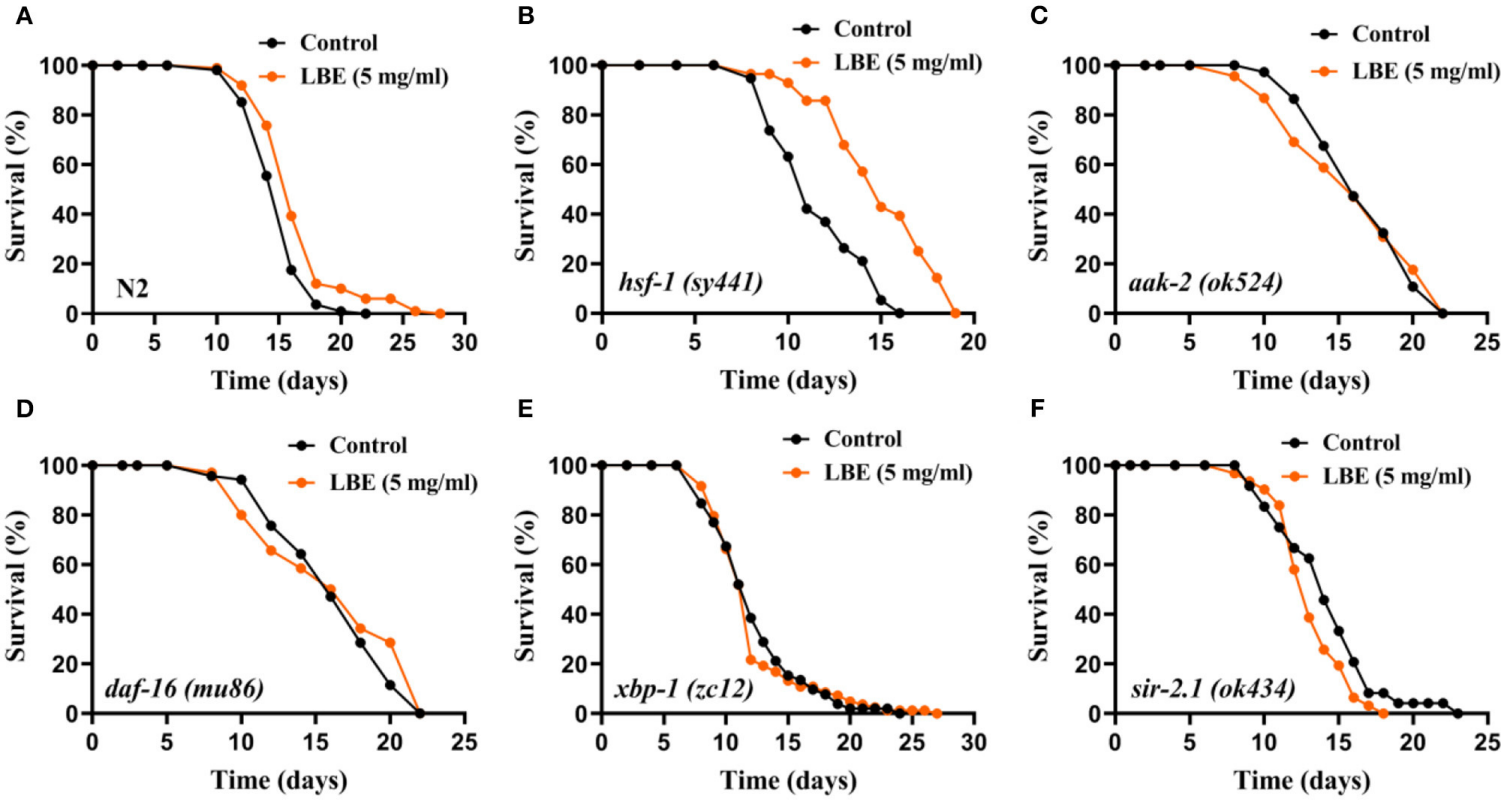
LBE still rescues *hsf-1* deficiency in mutant worms under favorable conditions. **(A)** Survival curves of N2 worms cultured with vehicle or 5 mg/ml LBE at 20°C (control: *n* = 108, mean lifespan 15.3 days; 5 mg/ml LBE: *n* = 99, mean lifespan 16.8 days; *p* < 0.0001). **(B)** Survival curves of *hsf-1 (sy4421)* worms cultured with vehicle or 5 mg/ml LBE at 20°C (control: *n* = 19, mean lifespan 11.6 days; 5 mg/ml LBE: *n* = 28, mean lifespan 15.0 days; *p* < 0.0001). **(C)** Survival curves of *aak-2 (ok524)* worms cultured with vehicle or 5 mg/ml LBE at 20°C (control: *n* = 74, mean lifespan 16.8 days; 5 mg/ml LBE: *n* = 68, mean lifespan 16.1 days; *p* = 0.910). **(D)** Survival curves of *daf-16 (mu86)* worms cultured with vehicle or 5 mg/ml LBE at 20°C (control: *n* = 70, mean lifespan 16.3 days; 5 mg/ml LBE: *n* = 58, mean lifespan 15.1 days; *p* = 0.299). **(E)** Survival curves of *xbp-1 (zc12)* worms cultured with vehicle or 5 mg/ml LBE at 20°C (control: *n* = 52, mean lifespan 12.3 days; 5 mg/ml LBE: *n* = 83, mean lifespan 12.1 days; *p* = 0.819). **(F)** Survival curves of *sir-2.1 (ok434)* worms cultured with vehicle or 5 mg/ml LBE at 20°C (control: *n* = 24, mean lifespan 14.1 days; 5 mg/ml LBE: *n* = 31, mean lifespan 13.2 days; *p* = 0.113). All the results are representative of at least two independent lifespan experiments. Log-rank test was used to assess significance (see also Supplementary Table 5). LBE, total water extracts of *L. barbarum* berry.

Because the effects of LBE on longevity are greatly influenced by the mean lifespan of worms, we further evaluated the effect of LBE on the above mutant strains such as *hsf-1 (sy441), aak-2 (ok524), daf-16 (mu86), xbp-1 (zc12)*, and *sir-2.1 (ok434)* under favorable conditions (Figure 5). The effect of LBE on the longevity of the nematode decreased to 11% under favorable conditions (Figure 5A). However, LBE showed better antiaging effect on *hsf-1 (sy441)* worms (+29%, Figure 5B) than under unfavorable condition (+14%, Figure 4B). Unlike the results under unfavorable conditions, LBE did not extend the mean lifespan in either *aak-2 (ok524), daf-16 (mu86), or xbp-1 (zc12)* mutant worms (Figures 5C–E). Consistent with the results under unfavorable conditions, *sir-2.1 (ok434)* worms treated with LBE also showed no lifespan extension (Figure 5F). The results indicated that LBE can rescue *hsf-1* deficiency in worms under both unfavorable and favorable conditions.

### LBE Rescues *hsf-1* Deficiency Depending on *sir-2.1*

**Figure 6:**
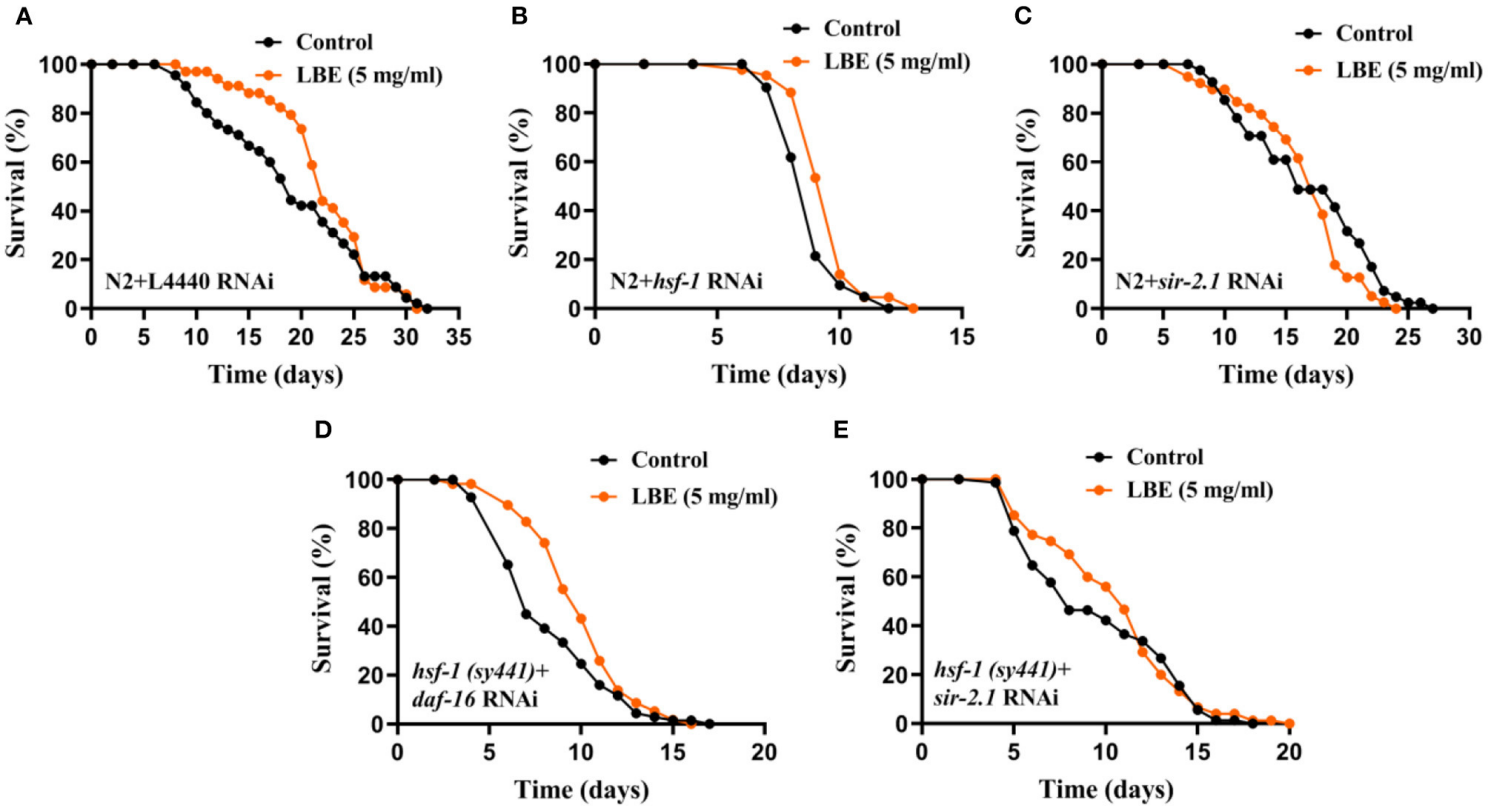
LBE rescues *hsf-1* deficiency depending on *sir-2.1*. **(A)** Lifespan analysis of N2 worms fed with empty vector L4440 RNAi bacteria treated with vehicle or 5 mg/ml LBE at 20°C (control: *n* = 45, mean lifespan 19.2 days; 5 mg/ml LBE: *n* = 34, mean lifespan 22.2 days; *p* = 0.256). **(B)** Lifespan analysis of N2 worms fed with *hsf-1* RNAi bacteria treated with vehicle or 5 mg/ml LBE at 20°C (control: *n* = 42, mean lifespan 8.9 days; 5 mg/ml LBE: *n* = 43, mean lifespan 9.6 days; *p* = 0.005). **(C)** Lifespan analysis of N2 worms fed with *sir-2.1* RNAi bacteria treated with vehicle or 5 mg/ml LBE at 20°C (control: *n* = 41, mean lifespan 17.0 days; 5 mg/ml LBE: *n* = 39, mean lifespan 16.6 days; *p* = 0.172). **(D)** Lifespan analysis of *hsf-1 (sy441)* worms fed with *daf-16* RNAi bacteria treated with vehicle or 5 mg/ml LBE at 20°C (control: *n* = 69, mean lifespan 8.3 days; 5 mg/ml LBE: *n* = 58, mean lifespan 9.9 days; *p* = 0.018). **(E)** Lifespan analysis of *hsf-1 (sy441)* worms fed with sir*-2.1* RNAi bacteria treated with vehicle or 5 mg/ml LBE at 20°C (control: *n* = 71, mean lifespan 9.6 days; 5 mg/ml LBE: *n* = 75, mean lifespan 10.6 days; *p* = 0.470). For all lifespan analysis, FUdR at 30 μM was supplemented to prevent progeny production. All the results are representative of at least two independent lifespan experiments. Log-rank test was used to assess significance (see also Supplementary Table 6). LBE, total water extracts of *L. barbarum* berry.

To verify the results that LBE rescues *hsf-1* deficiency, we performed RNAi experiments on N2 worms fed by empty vector L4440, *hsf-1*or *sir-2.1* RNAi bacteria. Although LBE had no effect on N2 worms with L4440 (Figure 6A) or *sir-2.1* (Figure 6C) RNAi, LBE still showed the antiaging effect on N2 worms with *hsf-1* RNAi (+8%, Figure 6B).

To further understand the mechanism of how LBE rescues *hsf-1* deficiency, we monitored the survival of *hsf-1 (sy441)* worms fed with *daf-16* or *sir-2.1* RNAi bacteria and treated with LBE or vehicle. We found that *hsf-1 (sy441)* worms silenced by *daf-16* RNAi still showed an extended mean lifespan with LBE treatment as compared to vehicle-treated group (+19%, Figure 6D), indicating that the effects of LBE on rescuing *hsf-1* deficiency are *daf-16-*independent. In contrast, there was no significant difference in mean lifespan between LBE and vehicle-treated groups in *hsf-1 (sy441)* worms silenced by *sir-2.1* RNAi (Figure 6E). Thus, the effect of LBE on rescuing *hsf-1* deficiency is *sir-2.1-*dependent.

### LBE Extends Lifespan and Alleviates Proteotoxicity in Neurodegenerative Worms With *hsf-1* Deficiency

**Figure 7:**
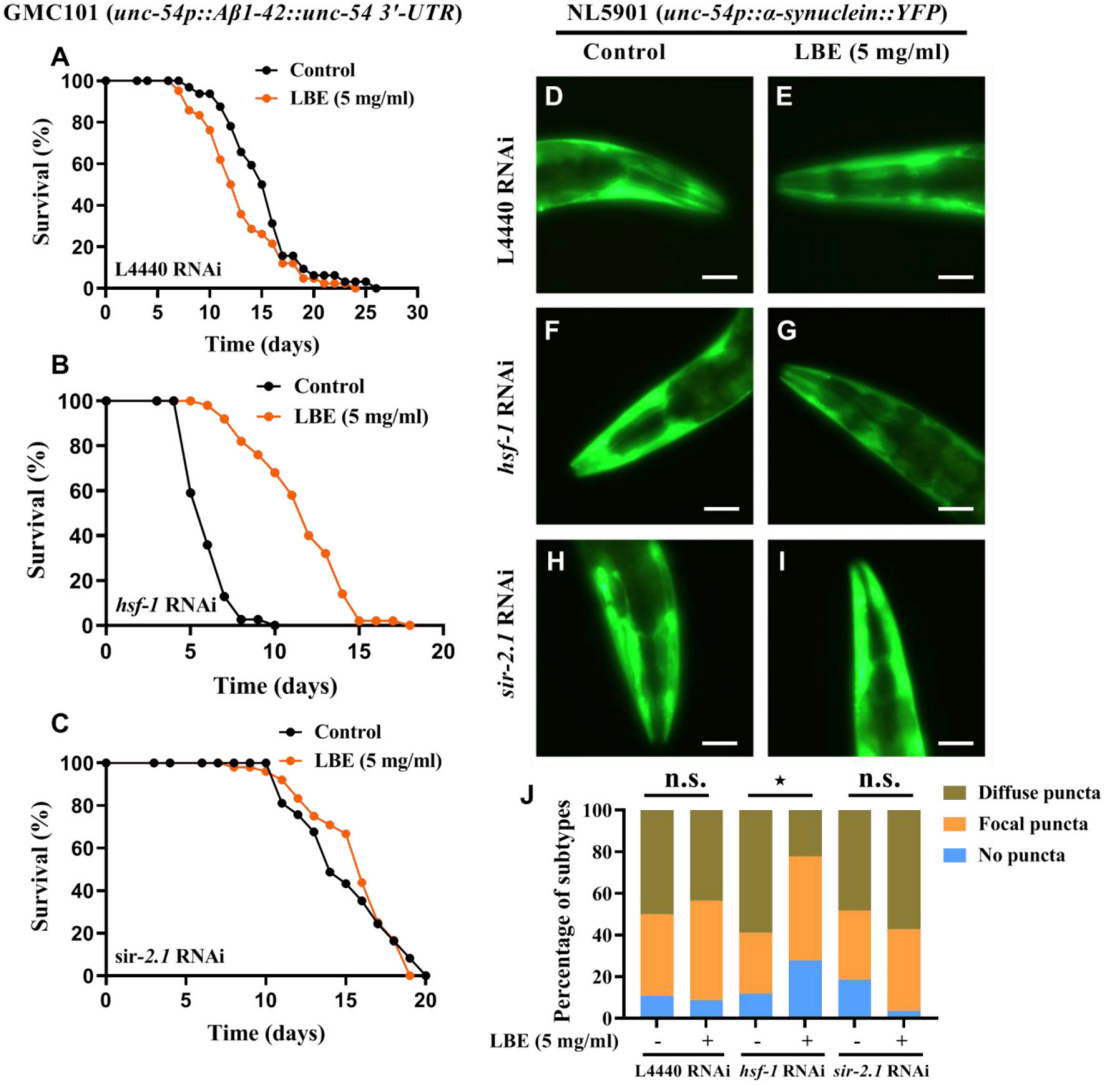
LBE extends lifespan and alleviates toxic protein aggregation in neurodegenerative worms with *hsf-1* deficiency. **(A)** Lifespan analysis of GMC101 (*unc-54p::A*β*1-42::unc-54 3'-UTR*) worms fed with empty vector L4440 RNAi bacteria treated with vehicle or 5 mg/ml LBE at 20°C (control: *n* = 32, mean lifespan 15.3 days; 5 mg/ml LBE: *n* = 42, mean lifespan 13.0 days; *p* = 0.051). **(B)** Lifespan analysis of GMC101 worms fed with *hsf-1* RNAi bacteria treated with vehicle or 5 mg/ml LBE at 20°C (control: *n* = 39, mean lifespan 6.1 days; 5 mg/ml LBE: *n* = 50, mean lifespan 11.7 days; *p* < 0.0001). **(C)** Lifespan analysis of GMC101 worms fed with *sir-2.1* RNAi bacteria treated with vehicle or 5 mg/ml LBE at 20°C (control: *n* = 37, mean lifespan 15.0 days; 5 mg/ml LBE: *n* = 48, mean lifespan 15.6 days; *p* = 0.715). **(D–I)** Graphical representations for epifluorescence visualization of α-synuclein aggregation in anterior region of NL5901 (*unc-54p::*α*-synuclein::YFP*) worms at day 3 (~20 worms per condition). **(D,E)**, NL5901 fed with empty vector L4440 RNAi bacteria in the absence or presence of 5 mg/ml LBE. **(F,G)**, NL5901 fed with *hsf-1* RNAi bacteria in the absence or presence of 5 mg/ml LBE. **(H,I)**, NL5901 fed with *sir-2.1* RNAi bacteria in the absence or presence of 5 mg/ml LBE. Scale bar, 50 μm. **(J)** Bar chart of classification (no puncta, focal puncta, or diffuse puncta according to the degree of α-synuclein aggregation on fluorescent graphs) composition for per condition. All the results are representative of at least two independent experiments. Log-rank test [for **(A–C)**] or Mann–Whitney *U* test [for **(J)**] was used to assess significance. n.s., not significant difference, ^⋆^*P* < 0.05 (see also Supplementary Table 7). LBE, total water extracts of *L. barbarum* berry.

It has been reported that *hsf-1* deficiency with aging is associated with neurodegenerative diseases (7). Since LBE can significantly rescue *hsf-1* deficiency in wild-type worms, we investigated the effects of LBE on neurodegenerative worms with *hsf-1* deficiency. We found that under favorable conditions, LBE significantly prolonged the lifespan of transgenic Aβ (1–42) GMC101 nematodes silenced by *hsf-1* RNAi (+92%), but it had little effect on GMC101 nematodes silenced by L4440 or *sir-2.1* RNAi (Figures 7A–C). We also examined the effect of LBE on α-synuclein aggregation in NL5901 nematodes, and the results showed that LBE alleviated α-synuclein puncta formation in NL5901 worms fed with *hsf-1* RNAi bacteria, but it did not reduce the protein aggregation in NL5901 worms fed with L4440 or *sir-2.1* RNAi bacteria (Figures 7D–J). These results indicated that LBE can extend lifespan and alleviate proteotoxicity in neurodegenerative worms with *hsf-1* deficiency.

## Discussion

**Figure 8:**
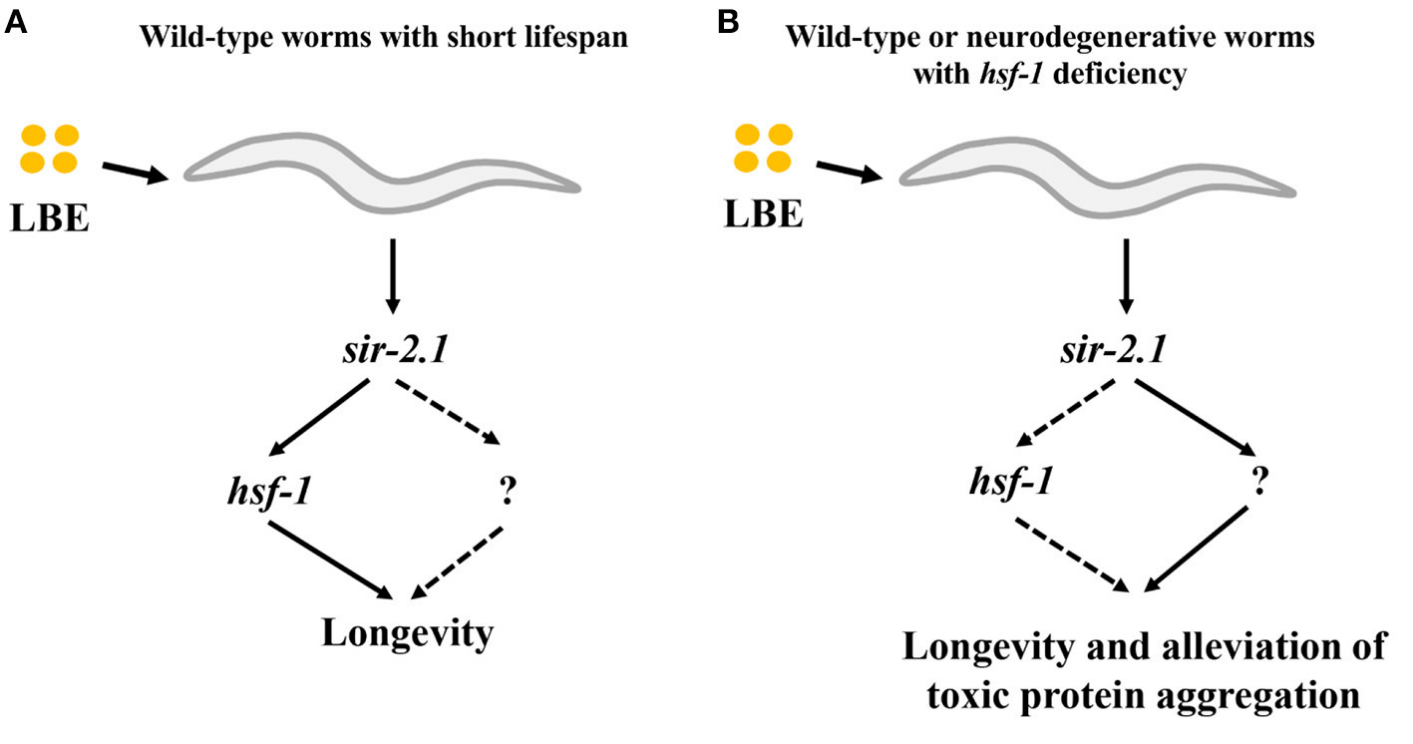
Model of LBE for lifespan extension and alleviation of toxic protein aggregation. **(A)** For wild-type worms with short lifespan, LBE shows the longevity effect depending on *sir-2.1* and *hsf-1* pathway. **(B)** For wild-type or neurodegenerative worms with *hsf-1* deficiency, LBE also extends the lifespan and alleviates toxic protein aggregation in a *sir-2.1*-dependent manner, possibly through an unknown downstream target. LBE, total water extracts of *L. barbarum* berry.

In this study, we demonstrate that the effect of LBE on longevity depends on the mean lifespan of worms. LBE has a better antiaging effect for worms cultured under unfavorable conditions as compared to those under favorable conditions, and this effect depends on *sir-2.1* (Figure 8A). Furthermore, LBE can rescue *hsf-1* deficiency, also depending on *sir-2.1* (Figure 8B).

For thousands of years, traditional Chinese medicine scriptures have documented the benefits of *L. barbarum* berry for lifespan extension. Although few study has reported that *L. barbarum* berry or LBP extends the lifespan of fruit flies and has no effect on the lifespan of mice (24, 27), the effects of *L. barbarum* berry on lifespan are needed to be investigated in more detail as lifespan is easily influenced by environmental and genetic factors (28–30). Meanwhile, the length of mean lifespan may affect the antiaging effect of *L. barbarum* berry. We found that the effect of LBE on longevity is negatively correlated with mean lifespan in *C. elegans*. In other words, LBE has a better antiaging effect on worms with shorter mean lifespan as compared to that with longer mean lifespan.

We also found that the antiaging effect of LBE under unfavorable conditions depends on *sir-2.1*. As a conserved protein deacetylase, sir-2.1 removes acetyl from cellular histone proteins through the coenzyme nicotinamide adenine dinucleotide (NAD^+^) (31, 32) and regulates the response to calorie restriction and increases lifespan (33, 34). Unfavorable treatment may result in a decreased NAD^+^ level with downregulated sir-2.1 activity, which shortens the lifespan of worms. LBE may increase the NAD^+^ level, activate sir-2.1 pathway, and therefore prolong the lifespan. Indeed, the function defect of *sir-2.1* in worms eliminates the antiaging effect of LBE. Therefore, we speculate that this may be one of the main antiaging mechanisms of LBE.

More importantly, LBE can rescue *hsf-1* deficiency in wild-type worms under either unfavorable or favorable conditions and also in neurodegenerative worms. Impairment of *hsf*-1 activity or expression in neurodegenerative diseases has been widely confirmed (35, 36). Thus, LBE may be a potential therapeutic drug for neurodegenerative diseases. Consistent with the antiaging effect of LBE, LBE rescues *hsf-1* deficiency also in a *sir-2.1*-dependent manner. As a protein deacetylase, SIRT1 can deacetylate transcriptional regulator *hsf*-1 (37) and also p53 (38), NF-κB (39), FOXOs (40), and PGC-1α (41). The downstream target genes of these transcription factors, such as *HSP70, BDNF, CASP3, BCL2*, and *SOD2*, are involved in a broad range of biological processes including protein quality control, cell survival, antioxidant, and antiinflammatory responses (42, 43). Thus, it will be interesting to study whether LBE can enhance the activity of other transcription factors and downstream molecules through SIRT1 to rescue *hsf-1* deficiency and extend lifespan.

## Conclusion

In the study, we found that longevity effect of LBE is negatively correlated with the mean lifespan in *C. elegans*. This implies that LBE may be a feasible antiaging natural dietary supplement especially to individuals with malnutrition or chronic diseases. We also found that LBE can rescue *hsf*-1 deficiency in wild-type and neurodegenerative worms. Thus, LBE may be a potential therapeutic agent for neurodegenerative diseases characterized by *hsf*-1 deficiency. It is inevitable that the study also has some limitations, such as lack of studies on longevity effects of other components of LBE including anthocyanins, which requires further study in the future.

## Data Availability Statement

The original contributions presented in the study are included in the article/Supplementary Material, further inquiries can be directed to the corresponding author/s.

## Author Contributions

HZ, C-cW, and LW performed conceptualization. C-cW and LW involved in funding acquisition, involved in writing, reviewing, and editing. HZ, JF, DY, and SD performed the investigation. CS and XW provided the resources. HZ wrote original draft. All authors have read and agreed to the published version of the manuscript.

## Funding

This study was funded by Ningxia Key Research and Development Program Grant (2016BZ05), National Key R&D Program of China (2017YFA0504000), and Youth Innovation Promotion Association, CAS, to LW.

## Conflict of Interest

The authors declare that the research was conducted in the absence of any commercial or financial relationships that could be construed as a potential conflict of interest.

## Publisher's Note

All claims expressed in this article are solely those of the authors and do not necessarily represent those of their affiliated organizations, or those of the publisher, the editors and the reviewers. Any product that may be evaluated in this article, or claim that may be made by its manufacturer, is not guaranteed or endorsed by the publisher.
