# Supplementary material for: Lycium barbarum Extracts Extend Lifespan and Alleviate Proteotoxicity in Caenorhabditis elegans
Source: Front Nutr. 2022 Jan 12;8:815947. doi: 10.3389/fnut.2021.815947 (PMC8790518; doi:10.3389/fnut.2021.815947)
Supplement: Supplementary file 1 [file Data_Sheet_1.PDF]

# *Supplementary Material*

## 1 Supplementary Figures

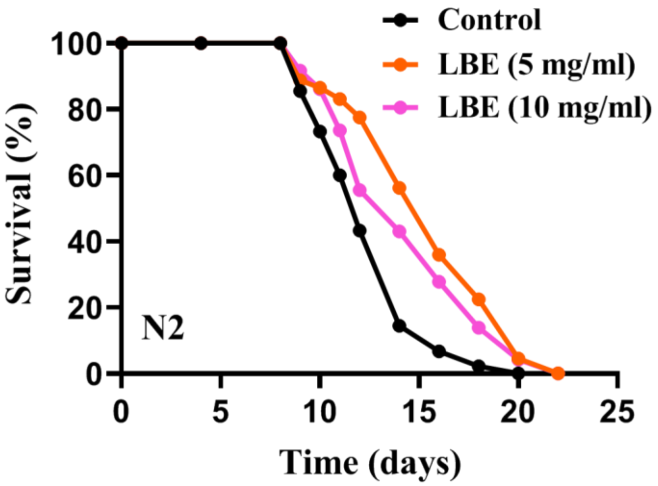


**Supplementary Figure 1.** LBE at 10 mg/ml had no better anti-aging effect than 5 mg/ml. Survival curves of N2 worms cultured under unfavorable conditions at 20°C with vehicle (5% water), 5 or 10 mg/ml LBE (control: n=90, mean lifespan 12.5 days; 5 mg/ml LBE: n=89, mean lifespan 15.5 days, *P*<0.0001 v.s. control; 10 mg/ml LBE: n=72, mean lifespan 13.6 days, *P*=0.081 v.s. 5 mg/ml LBE). LBE, total water extracts of *L. barbarum* berry.

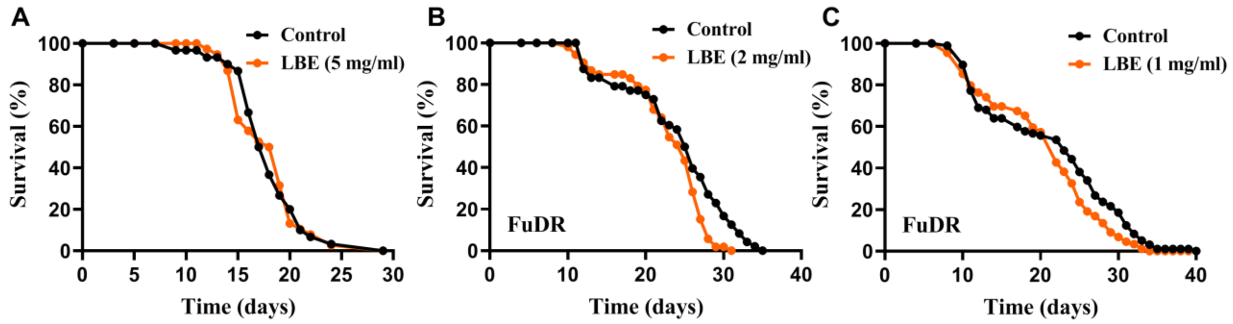


**Supplementary Figure 2.** LBE had no lifespan extension effect on worms with mean lifespan more than 17 days. (A) Survival curves of N2 worms treated with vehicle or 5 mg/ml LBE at 20°C (control: n=30, mean lifespan 17.9 days; 5 mg/ml LBE: n=38, mean lifespan 17.8 days, *P*=0.861). (B) Survival curves of N2 worms treated with vehicle or 2 mg/ml LBE at 20°C (control: n=48, mean lifespan 24.0 days; 5 mg/ml LBE: n=53, mean lifespan 22.8 days, *P*=0.016). (C) Survival curves of N2 worms treated with vehicle or 1 mg/ml LBE at 20°C (control: n=97, mean lifespan 21.2 days; 5 mg/ml LBE: n=89, mean lifespan 20.3 days, *P*=0.041). LBE, total water extracts of *L. barbarum* berry.

## 2 Supplementary Tables

**Supplementary Table 1.** Lifespan analysis of N2 worms treated with LBP at concentrations of 300 µg/ml, related to Figure 1A and B.

| Genotype | Condition | Mean lifespan (Days) | Std error (Days) | No. total | No. dead | No. censored | Culturing temperature | Mean lifespan extention (%) vs ctrl | Log rank test (*P*-value) vs ctrl |
|---|---|---|---|---|---|---|---|---|---|
| N2 | Control | 17.8 | 0.4 | 80 | 71 | 9 | 20℃ | | |
| | LBP (300 µg/ml) | 17.3 | 0.5 | 80 | 71 | 9 | 20℃ | -2.81 | 0.864 |
| N2 | Control | 9.7 | 0.3 | 130 | 89 | 41 | 25℃ | | |
| | LBP (300 µg/ml) | 9.7 | 0.3 | 130 | 108 | 22 | 25℃ | 0 | 0.898 |
| N2 | Control | 13.0 | 0.7 | 50 | 23 | 27 | 20℃ | | |
| | LBP (300 µg/ml) | 12.5 | 0.7 | 50 | 22 | 28 | 20℃ | -3.84 | 0.678 |

**Supplementary Table 2.** Lifespan analysis of N2 worms treated with LBE at concentrations of 1, 2 and 5 mg/ml, related to Figure 2B.

| Genotype | Condition | Mean lifespan (Days) | Std error (Days) | No. total | No. dead | No. censored | Culturing temperature | Mean lifespan extention (%) | Log rank test (*P*-value) vs ctrl |
|---|---|---|---|---|---|---|---|---|---|
| N2 | Control | 11.8 | 0.5 | 114 | 49 | 65 | 20℃ | | |
| | LBE (1 mg/ml) | 12.6 | 0.5 | 113 | 51 | 62 | 20℃ | 6.78 | 0.428 |
| | LBE (2 mg/ml) | 13.5 | 0.4 | 113 | 91 | 22 | 20℃ | 14.41 | 0.011 |
| | LBE (5 mg/ml) | 16.3 | 0.6 | 88 | 68 | 20 | 20℃ | 38.14 | <0.0001 |
| N2 | Control | 13.9 | 0.5 | 115 | 72 | 43 | 20℃ | | |
| | LBE (1 mg/ml) | 14.1 | 0.4 | 114 | 94 | 20 | 20℃ | 1.44 | 0.703 |
| | LBE (2 mg/ml) | 16.2 | 0.5 | 118 | 71 | 47 | 20℃ | 16.55 | 0.002 |
| | LBE (5 mg/ml) | 18.7 | 0.4 | 118 | 99 | 19 | 20℃ | 34.53 | <0.0001 |

**Supplementary Table 3.** Lifespan analysis of N2 worms with different mean lifespan treated with LBE at 5 mg/ml, related to Figure 3.

| Genotype | Condition | Mean lifespan (Days) | Std error (Days) | No. total | No. dead | No. censored | Culturing temperature | Mean lifespan extention (%) vs ctrl | Log rank test (*P*-value) |
|---|---|---|---|---|---|---|---|---|---|
| N2 | Control | 11.8 | 0.5 | 114 | 49 | 65 | 20℃ | | |
| | LBE (5 mg/ml) | 16.3 | 0.6 | 88 | 68 | 20 | 20℃ | 38.14 | <0.0001 |
| N2 | Control | 13.9 | 0.5 | 115 | 72 | 43 | 20℃ | | |
| | LBE (5 mg/ml) | 18.7 | 0.4 | 118 | 99 | 19 | 20℃ | 34.53 | <0.0001 |
| N2 | Control | 13.1 | 0.2 | 153 | 62 | 91 | 20℃ | | |
| | LBE (5 mg/ml) | 18.8 | 0.5 | 158 | 90 | 68 | 20℃ | 43.51 | <0.0001 |
| N2 | Control | 12.5 | 0.3 | 163 | 90 | 73 | 20℃ | | |
| | LBE (5 mg/ml) | 15.5 | 0.4 | 154 | 89 | 65 | 20℃ | 24.00 | <0.0001 |
| N2 | Control | 11.6 | 0.3 | 159 | 80 | 79 | 20℃ | | |
| | LBE (5 mg/ml) | 15.5 | 0.4 | 160 | 82 | 78 | 20℃ | 33.62 | <0.0001 |
| N2 | Control | 11.3 | 0.2 | 150 | 110 | 40 | 20℃ | | |
| | LBE (5 mg/ml) | 17.1 | 0.3 | 150 | 116 | 34 | 20℃ | 51.33 | <0.0001 |
| N2 | Control | 12.4 | 0.2 | 153 | 93 | 60 | 20℃ | | |
| | LBE (5 mg/ml) | 15.1 | 0.3 | 153 | 125 | 28 | 20℃ | 21.77 | <0.0001 |
| N2 | Control | 14.0 | 0.4 | 80 | 44 | 36 | 20℃ | | |

| | | | | | | | | | |
|---|---|---|---|---|---|---|---|---|---|
| | LBE (5 mg/ml) | 15.1 | 0.5 | 80 | 57 | 23 | 20℃ | 7.85 | 0.023 |
| Genotype | Condition | Mean lifespan (Days) | Std error (Days) | No. total | No. dead | No. censored | Culturing temperature | Mean lifespan extention (%) vs ctrl | Log rank test (*P*-value) |
| N2 | Control | 15.3 | 0.2 | 150 | 108 | 42 | 20℃ | | |
| | LBE (5 mg/ml) | 16.8 | 0.3 | 150 | 99 | 51 | 20℃ | 9.80 | <0.0001 |
| N2 | Control | 17.9 | 0.7 | 50 | 30 | 20 | 20℃ | | |
| | LBE (5 mg/ml) | 17.8 | 0.5 | 50 | 38 | 12 | 20℃ | -0.56 | 0.861 |

**Supplementary Table 4.** Lifespan analysis of N2 and mutant worms treated with LBE at 5 mg/ml under unfavorable conditions, related to Figure 4.

| Genotype | Condition | Mean lifespan (Days) | Std error (Days) | No. total | No. dead | No. censored | Culturing temperature | Mean lifespan extention (%) vs ctrl | Log rank test (p-value) vs ctrl |
|---|---|---|---|---|---|---|---|---|---|
| N2 | Control | 11.6 | 0.3 | 159 | 80 | 79 | 20℃ | | |
| | LBE (5 | 15.5 | 0.4 | 160 | 82 | 78 | 20℃ | 33.62 | <0.0001 |
| *hsf-1(sy441)* | Control | 9.7 | 0.2 | 164 | 72 | 92 | 20℃ | | |
| | LBE (5 | 11.1 | 0.2 | 167 | 97 | 70 | 20℃ | 14.43 | <0.0001 |
| *aak-2(ok524)* | Control | 13.4 | 0.3 | 153 | 100 | 53 | 20℃ | | |
| | LBE (5 | 17.1 | 0.3 | 152 | 114 | 38 | 20℃ | 27.61 | <0.0001 |
| *daf-16(mu86)* | Control | 10.8 | 0.2 | 150 | 86 | 64 | 20℃ | | |
| | LBE (5 | 16.6 | 0.4 | 150 | 90 | 60 | 20℃ | 53.70 | <0.0001 |
| *xbp-1(zc12)* | Control | 12.7 | 0.3 | 165 | 61 | 104 | 20℃ | | |
| | LBE (5 | 17.3 | 0.4 | 163 | 80 | 83 | 20℃ | 36.22 | <0.0001 |
| *sir-2.1(ok434)* | Control | 10.8 | 0.2 | 150 | 119 | 31 | 20℃ | | |
| | LBE (5 | 10.6 | 0.4 | 150 | 63 | 87 | 20℃ | -1.85 | 0.981 |

**Supplementary Table 5.** Lifespan analysis of N2 and mutant worms treated with LBE at 5 mg/ml under favorable conditions, related to Figure 5.

| Genotype | Condition | Mean lifespan (Days) | Std error (Days) | No. total | No. dead | No. censored | Culturing temperature | Mean lifespan extention (%) vs ctrl | Log rank test (p-value) vs ctrl |
|---|---|---|---|---|---|---|---|---|---|
| N2 | Control | 15.3 | 0.2 | 150 | 108 | 42 | 20℃ | | |
| | LBE (5 | 16.8 | 0.3 | 150 | 99 | 51 | 20℃ | 10.46 | <0.0001 |
| *hsf-1(sy441)* | Control | 11.6 | 0.6 | 50 | 19 | 31 | 20℃ | | |
| | LBE (5 | 15.0 | 0.6 | 50 | 28 | 22 | 20℃ | 29.31 | <0.0001 |
| *aak-2(ok524)* | Control | 16.8 | 0.4 | 120 | 74 | 46 | 20℃ | | |
| | LBE (5 | 16.1 | 0.5 | 120 | 68 | 52 | 20℃ | -4.17 | 0.91 |
| *daf-16(mu86)* | Control | 16.3 | 0.5 | 120 | 70 | 50 | 20℃ | | |
| | LBE (5 | 15.1 | 0.6 | 120 | 58 | 62 | 20℃ | -7.36 | 0.299 |
| *xbp-1(ok434)* | Control | 12.3 | 0.5 | 100 | 52 | 48 | 20℃ | | |
| | LBE (5 | 12.1 | 0.4 | 100 | 83 | 17 | 20℃ | -1.63 | 0.819 |
| *sir-2.1(ok434)* | Control | 14.1 | 0.7 | 50 | 24 | 26 | 20℃ | | |
| | LBE (5 | 13.2 | 0.4 | 50 | 31 | 19 | 20℃ | -6.38 | 0.113 |

**Supplementary Table 6.** Lifespan analysis of N2 and *hsf-1(sy441)* worms treated with indicated RNAi and/or LBE at 5 mg/ml, related to Figure 6.

| Genotype | Condition | Mean lifespan (Days) | Std error (Days) | No. total | No. dead | No. censored | Culturing temperature | Mean lifespan extention (%) vs ctrl | Log rank test (p-value) vs ctrl |
|---|---|---|---|---|---|---|---|---|---|
| N2 | l4440-control | 19.2 | 1.0 | 60 | 45 | 15 | 20℃ | | |
| | l4440-LBE (5 mg/ml) | 22.2 | 0.9 | 60 | 34 | 26 | 20℃ | 15.63 | 0.256 |
| | *hsf-1* RNAi-control | 8.9 | 0.2 | 60 | 42 | 18 | 20℃ | | |
| | *hsf-1* RNAi-LBE (5 mg/ml) | 9.6 | 0.2 | 60 | 43 | 17 | 20℃ | 7.87 | 0.005 |
| | *sir-2.1* RNAi-control | 17.0 | 0.8 | 60 | 41 | 19 | 20℃ | | |
| | *sir-2.1* RNAi-LBE (5 | 16.6 | 0.7 | 60 | 39 | 21 | 20℃ | -2.35 | 0.172 |
| *hsf-1 (sy441)* | *daf-16* RNAi-control | 8.3 | 0.4 | 80 | 69 | 11 | 20℃ | | |
| | *daf-16* RNAi-LBE (5 | 9.9 | 0.3 | 80 | 58 | 22 | 20℃ | 19.28 | 0.018 |
| | *sir-2.1* RNAi-control | 9.6 | 0.5 | 80 | 71 | 9 | 20℃ | | |
| | *sir-2.1* RNAi-LBE (5 | 10.6 | 0.4 | 80 | 75 | 5 | 20℃ | 10.42 | 0.47 |

**Supplementary Table 7.** Lifespan analysis of GMC101 worms treated with indicated RNAi and/or LBE at 5 mg/ml, related to Figure 7.

| Genotype | Condition | Mean lifespan (Days) | Std error (Days) | No. total | No. dead | No. censored | Culturing temperature | Mean lifespan extention (%) vs ctrl | Log rank test (p-value) vs ctrl |
|---|---|---|---|---|---|---|---|---|---|
| GMC101 | l4440-control | 15.3 | 0.7 | 60 | 32 | 28 | 20℃ | | |
| | l4440-LBE (5 mg/ml) | 13.0 | 0.6 | 60 | 42 | 18 | 20℃ | -15.03 | 0.051 |
| | *hsf-1* RNAi-control | 6.1 | 0.2 | 60 | 39 | 21 | 20℃ | | |
| | *hsf-1* RNAi-LBE (5 mg/ml) | 11.7 | 0.4 | 60 | 50 | 10 | 20℃ | 92.80 | <0.0001 |
| | *sir-2.1* RNAi-control | 15.0 | 0.5 | 60 | 37 | 23 | 20℃ | | |
| | *sir-2.1* RNAi-LBE (5 | 15.6 | 0.4 | 60 | 48 | 12 | 20℃ | 0.04 | 0.715 |
